# Unusual localization and presentation of osteoid osteoma mimicking juvenile spondyloarthritis: a case report

**DOI:** 10.1186/s12891-018-2383-1

**Published:** 2019-01-08

**Authors:** Josip Vlaic, Lovro Lamot, Sven Simunic, Miroslav Harjacek, Davor Bojic

**Affiliations:** 1 Division of Paediatric Orthopaedic Surgery, Children’s Hospital Zagreb, Ulica Vjekoslava Klaica 16, 10000 Zagreb, Croatia; 2 Division of Clinical Immunology and Rheumatology, Department of Paediatrics, Sestre milosrdnice University Hospital Centre, Vinogradska cesta, 29 Zagreb, Croatia; 3 Department of Paediatrics, University of Zagreb School of Medicine, Salata 3, Zagreb, Croatia; 4 Josip Juraj Strossmayer University of Osijek - Faculty of Medicine, Ulica cara Hadrijana 10e, 31000 Osijek, Croatia; 5 Josip Juraj Strossmayer University of Osijek - Faculty of Dental Medicine and Health, Crkvena 21, 31000 Osijek, Croatia

**Keywords:** Dactylitis, Sausage digit, Juvenile spondyloarthritis, Distal phalanx osteoid osteoma

## Abstract

**Background:**

Osteoid osteoma is a painful benign skeletal tumour of unknown aetiology. Most often it occurs in the long bones of extremities and responds well to nonsteroidal anti-inflammatory medications. However, unusual localization and atypical presentation of this tumour might present a diagnostic challenge, especially if symptoms mimic that indicative of juvenile spondyloarthritis.

**Case presentation:**

A misdiagnosed ten-and-a-half-year-old girl with osteoid osteoma involving the distal phalanx of a little finger is presented. Her initial symptoms were pain and swelling of the little finger resembling dactylitis, while various imaging modalities showed signs of tenosynovitis, indicating a possible development of juvenile spondyloarthritis. Several trials of different non-steroid anti-inflammatory drugs gave no satisfactory results and ultrasound guided triamcinolone-hexacetonide injection provided only a short relief. Finally, almost three years after initial presentation, persistent clinical symptoms warranted repeated imaging that raised suspicion of an osteoid osteoma. Directed treatment with surgical intervention led to almost immediate and complete resolution of her symptoms.

**Conclusions:**

Osteoid osteoma should be suspected in case of a tender swelling of a digit in children and adolescents, regardless of initial imaging findings and clinical presentation. Early diagnosis and treatment of this benign condition can have a substantial impact on quality of life of patients and their families and protect them from many unnecessary diagnostic procedures and treatment.

**Electronic supplementary material:**

The online version of this article (10.1186/s12891-018-2383-1) contains supplementary material, which is available to authorized users.

## Background

Osteoid osteoma (OO) is the third most common benign bone tumour typically found in children, adolescents, and young adults between 10 and 35 years of age [1, 2]. It accounts for approximately 3% of all primary bone tumours, and has a strong male predilection (male to female ratio 3:1) [3]. Histologically it is characterized by a network of dilated vessels, osteoblasts, osteoid, and woven bone which comprises a central nidus surrounded by a rim of reactive osteosclerosis [3]. Radiographically, a lytic lucent area less than 1.5 cm in diameter is most commonly demonstrated, making it hard to notice, especially in the early stage of the disease [1, 4]. The most common localization of such lesions are the long bones of lower limbs, while OO in the distal finger phalanx has been rarely reported [5–22]. The typical clinical presentation of OO includes progressively increasing pain that is worse at night and responsive to non-steroid anti-inflammatory drugs (NSAIDs). Nevertheless, substantial variations have been described, causing a delay in diagnosis, inadequate treatment, and persistence of symptoms, particularly when regions other than the lower limbs are involved [17, 23]. With fingers affected, the clinical features might mimic dactylitis, sausage-like tender swelling of one or more digits extending beyond the joint margin [24]. Commonly, dactylitis is associated with juvenile spondyloarthritis, group of chronic rheumatic diseases seen in paediatric population more often than any of the bone tumours [25].

The aim of our report is to address the diagnostic pitfalls observed in atypical OO and emphasize OO as a possible cause of long-lasting symptoms resembling those seen in juvenile spondyloarthritis.

## Case presentation

**Fig. 1:**
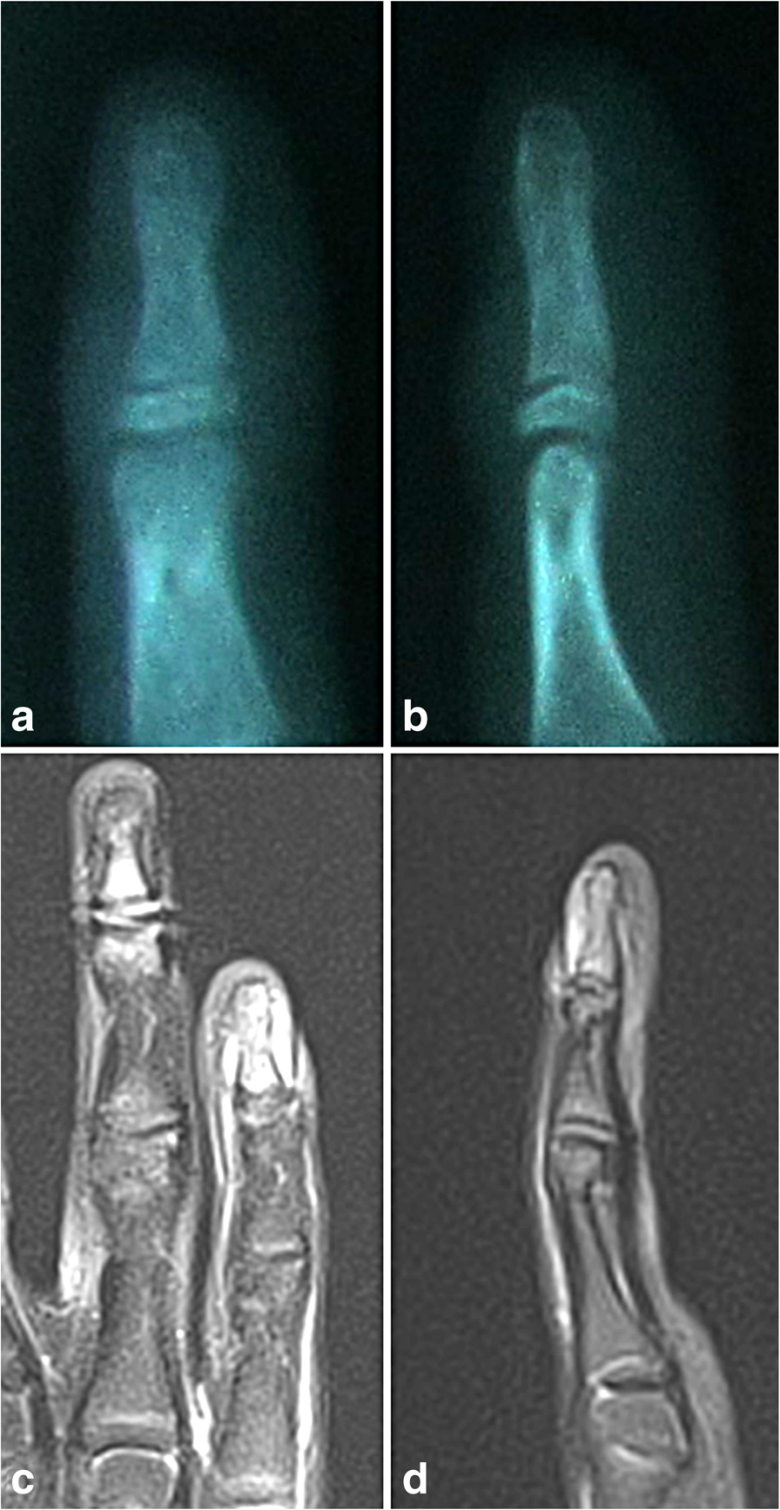
Initial radiographs of the left small finger distal phalanx in the anteroposterior (**a**) and lateral (**b**) view. Bone structure and mineralization is unremarkable and no signs of pathology are present. Magnetic resonance imaging of the left little finger in proton density fast spin echo sequence in the coronal (**c**) and sagittal (**d**) plane. Signal hyperintensity in the area of the distal phalanx, with no bone lesions is observed. These findings correlate with soft-tissue oedema medial, lateral and dorsal to distal phalanx

**Fig. 2:**
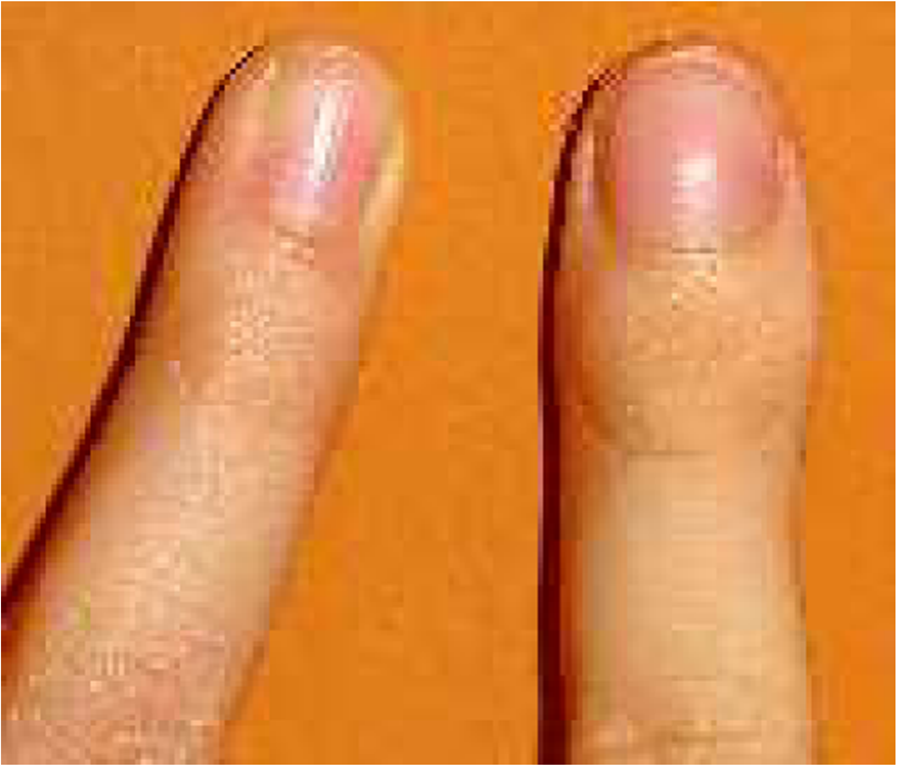
A preoperative photograph comparing distal part of the right and left small finger in dorsal view. On the left small finger, distal phalanx swelling and increase in the size of the nail bed give the appearance of a “sausage digit”, or dactylitis

**Fig. 3:**
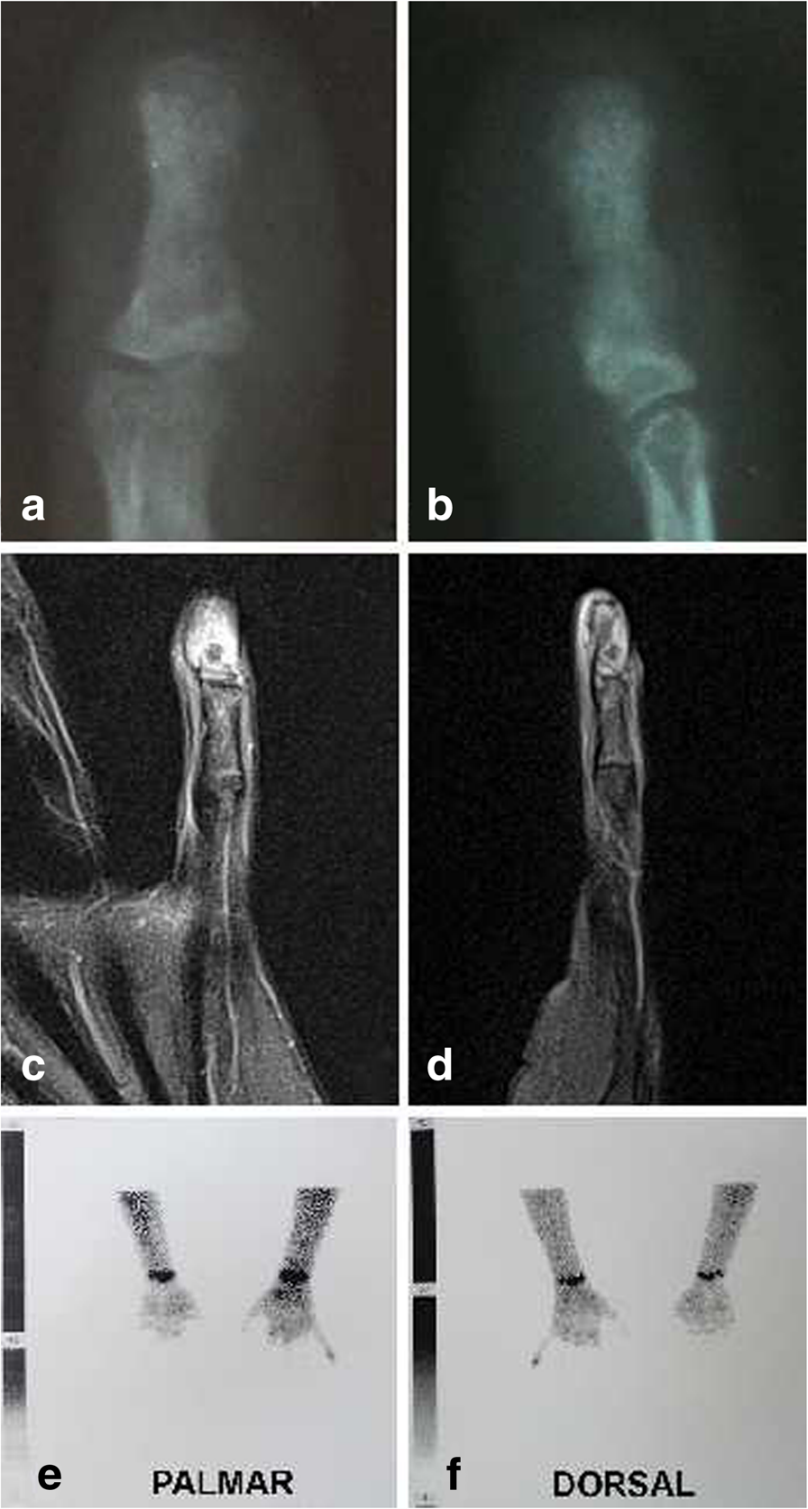
A follow up radiographs of the left small finger distal phalanx in anteroposterior (**a**) and lateral (**b**) view obtained two years after the symptoms begun. Diaphyseal distal phalanx widening and a central radiolucent zone are observed. Magnetic resonance image of the left small finger in proton density fast spin echo sequence in coronal (**c**) and sagittal (**d**) plane. Signal hyperintensity in the area of the distal phalanx that correlate with soft-tissue oedema is observed. On the dorsal aspect of the affected phalanx, a hypointensive oval lesion with no signs of cortical destruction is observed. These findings resemble an osteoid osteoma or sequestrum. An image of hands radionuclide skeletal scintigraphy with Technetium 99 m-MDP report in two projections palmar (**e**) and dorsal (**f**). Increased radionuclide uptake in distal phalanx/interphalangeal joint of the affected finger is predominantly observed in the dorsal projection

A ten-and-a-half-year-old girl presented to her primary care physician with a three-month history of swelling and pain in the distal part of the small finger of her left hand. The pain was most intense during movement and palpation, although occasionally it was present at rest as well. There was no history of preceding trauma, acute infection, or fever. Initial physical examination showed a thickened distal phalanx of the affected finger without motion restrictions. Initial radiograph showed normal bone structure and mineralization, without signs of fracture or other pathology (Fig. 1 a and b), and primary care physician suggested activity restriction. In the following five months, the pain became more prominent, without daily variations, and she was referred to a paediatric orthopaedic surgeon who suspected glomus tumour and ordered a magnetic resonance imaging (MRI) of the affected finger along with expanded laboratory workup. All laboratory findings, including CBC, CRP, ESR, rheumatoid factor, and antinuclear antibodies, were within the normal range. MRI showed a hyperintense signal on proton density fast spin echo sequence correlating with soft-tissue swelling surrounding distal phalanx (Fig. 1 c and d). These features were characterized by the radiologist as trauma or tenosynovitis. Ibuprofen trial was recommended and the patient initially reported slight reduction of swelling and pain, soon followed by subsequent deterioration. Finally, paediatric rheumatologist was consulted. The initial musculoskeletal ultrasound (MSUS) examination showed increased power-Doppler activity in the distal part of the affected finger with no effusion in the distal interphalangeal joint (DIP), and a cyst-like formation connected to the extensor tendon, giving the impression of tenosynovitis. Laboratory workup remained unremarkable. Due to persistent clinical and imaging findings suggestive of dactylitis, the diagnosis of juvenile spondyloarthritis was suspected. MSUS-guided triamcinolone-hexacetonid injection was administered in the cyst. The injection, along with an oral indomethacin trial, resulted in a slight reduction of the swelling. Nevertheless, the pain persisted, affecting the quality of sleep and activities of daily living. Short courses of various other NSAIDs were attempted, with no satisfactory results, so another consultation with paediatric orthopaedic surgeon was requested, nearly two years after the initial presentation. Clinical symptoms were still suggestive of dactylitis, with distal phalanx swelling and associated increase in size of the nail bed with pain on palpation (Fig. 2). Again, distal phalanx neoplasm was suspected and new radiographs and MRI were ordered, along with radionuclide skeletal scintigraphy with Technetium 99 m-MDP. At this point, two years after the initial radiographs, the newly-obtained radiographs of the left fifth finger showed diaphyseal widening of the distal phalanx with a central radiolucent zone. MRI findings were consistent with the radiographs and showed a small oval-shaped sequestrum measuring 3 mm in diameter on the dorsal aspect of the distal phalanx, along with the initially-described hyperintensity of the surrounding tissue. Radionuclide skeletal scintigraphy with Technetium 99 m-MDP showed increased radionuclide uptake in the distal phalanx/DIP joint of the affected finger in all three phases of the bone scan, a finding most typical for juvenile spondyloarthritis (Fig. 3 a to f). OO was finally suspected. After thorough preoperative planning, the patient underwent surgical exploration and excisional biopsy. A longitudinal skin incision was made over the dorsal aspect of the distal phalanx of the left small finger. The distal interphalangeal joint was exposed together with the extensor tendon insertion, which demonstrated the presence of inflammatory changes. In order to better visualize the distal phalanx, the nail plate was removed. The dorsal aspect of the distal phalanx was clinically inflamed with prominent red discolouration in an area measuring 4 mm in diameter. Following the bone corticotomy, a complete excisional biopsy of this area was performed, with specimen sent for pathological examination. The early postoperative course was uneventful with almost immediate pain relief in the finger. Pathology report returned positive for OO, describing the nidus within bony trabeculae surrounded by fibrovascular connective tissue. Three months after the surgery, the patient was pain-free with full range of motion of the involved finger. At the most recent four year postoperative follow up, there was no evidence of local recurrence, and the patient was symptom-free.

## Discussion and conclusions

The presented case of OO at an unusual localization with an atypical presentation took almost three years to be correctly diagnosed and treated (Additional file 1). In the majority of cases, OO is much easier to diagnose based on localization and clinical presentation. However, without typical signs and symptoms it may present a great burden to both the patient and family as well as the physicians involved. Clinically, a painful fingertip swelling may represent a heterogenous group of differential diagnoses including but not limited to trauma, whitlow or paronychia, osteomyelitis, glomus tumour, subungual exostosis, arthritis, soft tissue rheumatism (e.g. tenosynovitis), and OO [11, 15, 26]. Therefore, the average delay of more than two years between the onset of symptoms and the first special investigations have been reported in cases of OO in the distal finger phalanx [11].

While uncommon, other cases of OO of the distal phalanx have been presented in children around 10 years of age. However, all cases share similar clinical presentations with nail hypertrophy, local enlargement and night pain as the main symptoms [6–10]. In our case, OO caused pain and swelling of distal finger phalanx that extended beyond the borders of the osseous structures, giving an appearance of a “sausage digit”, commonly referred as dactylitis. This finding is not very common in children, and when tuberculosis, infection or sickle cell crisis are excluded in majority of cases dactylitis is associated with juvenile spondyloarthritis [27–29].

In our patient, three imaging modalities (MSUS, MRI, skeletal scintigraphy) demonstrated characteristic signs indicative of juvenile spondyloarthritis, although no other joint and/or enthesis was involved, and despite no family history of HLA-B27-associated diseases. Therefore, after several years of follow up with persistent symptoms, and without adequate response to NSAIDs or intraarticular glucocorticoids, re-evaluation by a second paediatric orthopaedics was suspicious for another pathology. Finally, three years after the initial symptoms and various, mostly inadequate treatment trials, repeated imaging led to the diagnosis of OO of the distal phalanx as a possible cause of the disturbances.

Although OO and rheumatic diseases have almost completely different underlying mechanisms, they do both share excessive production of prostaglandins at the affected site and therefore benefit from NSAIDs treatment. Nevertheless, several reports demonstrated that conservative management with NSAIDs may take years before the OO is resolved [30–32]. Therefore, it is not surprising our patient did not achieve sustained remission with this treatment modality.

The other dilemma elicited in our case is the sensitivity and specificity of various imaging modalities in the assessment of OO. While in the vast majority of cases OO can be easily identified with plain radiographs, computed tomography (CT) scans, MRI and/or skeletal scintigraphy, there are some areas of the skeleton, including spine, femoral neck and small bones of hands and feet, that are difficult to assess [33, 34]. Although dynamic contrast-enhanced MRI has been suggested as being useful to detect and to differentiate this tumour from OO-mimicking lesions, longstanding literature still supports thin-section CT as the most specific imaging study [17, 35, 36]. Besides the unexpected localization, the small size of the tumour (typically less-than 1.5–2 cm) could also be a cause of misdiagnosis [37]. Finally, recent literature also gives an argument in favour of the diagnostic riddle concerning atypical OO, with major issues being prolonged impairment and overtreatment [37].

In conclusion, we would like to emphasize OO as a possible cause of a tender swelling of a digit, a symptom more commonly seen in juvenile spondyloarthritis. Although OO is a benign disease, it might represent a diagnostic and treatment challenge for paediatric rheumatologists, indicating the need for consultation with other specialists, including the paediatric orthopaedics.

## Additional file

Additional file 1: Timeline for case report entitled “Unusual localization and presentation of osteoid osteoma mimicking juvenile spondyloarthritis: a case report.” (PDF 53 kb)

## Abbreviations

CRP: C reactive protein
CT: Computed tomography
DIP: Distal interphalangeal joint
ESR: Estimated sedimentation rate
MRI: Magnetic resonance imaging
MSUS: Musculoskeletal ultrasound
NSAIDs: Nonsteroidal anti-inflammatory drugs
OO: Osteoid osteoma
Technetium 99 m-MDP: Technetium 99 m-methyl diphosphonate

## References

[1] Johnson T, Steinbach L. Essentials of musculoskeletal imaging. 1st ed. Illinois: American Academy of Orthopedic Surgeons; 2004.

[2] Goodman CG, Fuller KS. Pathology: Implications for the Physical Thearpist, 4th Edition. St. Louis, Missouri: Saunders Elsevier; 2014.

[3] Folpe AL, Inwards CY (2010). Bone and soft tissue Pathology: a volume in the diagnostic Pathology series.

[4] Atesok KI, Alman BA, Schemitsch EH, Peyser A, Mankin H (2011). Osteoid osteoma and osteoblastoma. J Am Acad Orthop Surg.

[5] Sevitt S, Horn JS (1954). A painless and calcified osteoid osteoma of the little finger. J Pathol Bacteriol.

[6] Rosborough D (1966). Osteoid osteoma. Report of a lesion in the terminal phalanx of a finger. J Bone Joint Surg Br.

[7] Norman A, Dorfman HD (1975). Osteoid-osteoma inducing pronounced overgrowth and deformity of bone. Clin Orthop Relat Res.

[8] Giannikas A, Papachristou G, Tiniakos G, Chrysafidis G, Hartofilakidis-Garofalidis G (1977). Osteoid osteoma of the terminal phalanges. Hand.

[9] Levy Y, Rosenheck S, Greiff M, Torok G (1979). Osteoid osteoma of the distal phalanx of the thumb. Acta Orthop Scand.

[10] Bowen CV, Dzus AK, Hardy DA (1987). Osteoid osteomata of the distal phalanx. J Hand Surg Br.

[11] Foucher G, Lemarechal P, Citron N, Merle M (1987). Osteoid osteoma of the distal phalanx: a report of four cases and review of the literature. J Hand Surg Br..

[12] McCarten GM, Dixon PL, Marshall DR (1987). Osteoid osteoma of the distal phalanx: a case report. J Hand Surg Br..

[13] De Smet L, Fabry G (1996). Clubbing of single digit: an unusual cause. Clin Rheumatol.

[14] Marcuzzi A, Acciaro AL, Landi A (2002). Osteoid osteoma of the hand and wrist. J Hand Surg Br..

[15] Burger IM, McCarthy EF (2004). Phalangeal osteoid osteomas in the hand: a diagnostic problem. Clin Orthop Relat Res.

[16] Di Gennaro GL, Lampasi M, Bosco A, Donzelli O (2008). Osteoid osteoma of the distal thumb phalanx: a case report. Chir Organi Mov.

[17] Becce F, Jovanovic B, Guillou L, Theumann N (2011). Painful fingertip swelling of the middle finger. Osteoid osteoma of the distal phalanx of the middle finger. Skelet Radiol.

[18] Andalib A, Sajadie-Khajouei S (2013). Osteoid osteoma of distal phalanx: a rare disorder and review of literature. J Res Med Sci.

[19] Sonntag J, Engelund D (2014). Osteoid osteoma in the distal phalanx of the thumb. Ugeskr Laeger.

[20] Barbaric K, Prutki M, Starcevic D, Seiwerth S, Bojanic I (2016). Rare localization of osteoid osteoma - distal phalanx of the ring finger. Acta Med Croatica.

[21] Durgia B, Jain A, Agarwal S (2016). Osteoid osteoma of distal phalanx of middle finger-a diagnostic dilemma. J Hand Surg Asian Pac Vol..

[22] Horiuchi K, Horiuchi Y, Ochi K Osteoid osteoma of the distal phalanx of the ring finger with clubbed finger deformity: a case report. J Hand Surg Asian Pac Vol 2017;22:248–250. 10.1142/S0218810417720170 28506170

[23] Mungo DV, Zhang X, O'Keefe RJ, Rosier RN, Puzas JE, Schwarz EM (2002). COX-1 and COX-2 expression in osteoid osteomas. J Orthop Res.

[24] Kaeley GS, Eder L, Aydin SZ, Giuiterrez M, Bakewel C (2018). Dactylitis : a hallmark of psoriatic arthritis. Semin Arthritis Rheum.

[25] Colbert RA (2010). Classification of juvenile spondyloarthritis: Enthesitis-related arthritis and beyond. Nat Rev Rheumatol.

[26] Jones SN, Stoker DJ (1988). Radiology at your fingertips; lesions of the terminal phalanx. Clin Radiol.

[27] Stoll ML, Zurakowski D, Nigrovic LE, Nichols DP, Sundel RP, Nigrovic PA (2006). Patients with juvenile psoriatic arthritis comprise two distinct populations. Arthritis Rheum.

[28] Brousse V, Makani J, Rees DC (2014). Management of sickle cell disease in the community. BMJ.

[29] Rao GN, Gali JH, Tuberculous Dactylitis RSN (2016). An uncommon presentation of a common infection. Case Reports in Pediatrics.

[30] Kneisl JS, Simon MA (1992). Medical management compared with operative treatment for osteoid-osteoma. J Bone Joint Surg Am.

[31] Bottner F, Roedl R, Wortler K, Grethen C, Winkelmann W, Lindner N (2001). Cyclooxygenase-2 inhibitor for pain management in osteoid osteoma. Clin Orthop Relat Res.

[32] Leicester AW, Trantalis JN (2001). Osteoid osteoma in a young child: successful non-operative management. ANZ J Surg.

[33] Kayser F, Resnick D, Haghighi P, Pereira Edo R, Greenway G, Schweitzer M, Kindynis P (1998). Evidence of the subperiosteal origin of osteoid osteomas in tubular bones: analysis by CT and MR imaging. AJR Am J Roentgenol.

[34] Spouge AR, Thain LM (2000). Osteoid osteoma: MR imaging revisited. Clin Imaging.

[35] Hosalkar HS, Garg S, Moroz L, Pollock A, Dormans JP (2005). The diagnostic accuracy of MRI versus CT imaging for osteoid osteoma in children. Clin Orthop Relat Res.

[36] Amrani KK, Berger RA (2006). Radiology corner: osteoid osteoma of the index finger: case presentation and discussion. J Hand Surg Am.

[37] Ciftdemir M, Tuncel SA, Usta U (2015). Atypical osteoid osteomas. Eur J Orthop Surg Traumatol.

