# Supplementary material for: Unusual localization and presentation of osteoid osteoma mimicking juvenile spondyloarthritis: a case report
Source: BMC Musculoskelet Disord. 2019 Jan 8;20:17. doi: 10.1186/s12891-018-2383-1 (PMC6323825; doi:10.1186/s12891-018-2383-1)
Supplement: Supplementary file 1 — Timeline for case report entitled “Unusual localization and presentation of osteoid osteoma mimicking juvenile spondyloarthritis: a case report.” (PDF 53 kb) [file 12891_2018_2383_MOESM1_ESM.pdf]

Dates	Summaries from Initial and Follow-up Visits	Diagnostic Testing (including dates)	Interventions
**06/02/2012**	Initial visit to GP	Hand radiograms	
**25/04/2012**	Visit to physical medicine and rehabilitation specialist (rheumatology department)	Initial visit	Physical therapy, consultation of paediatric orthopaedic surgeon requested
**09/05/2012**	Visit to paediatric orthopaedic surgeon	Initial visit, Blood testing (SR, CBC, CRP, AP, RF and ANF , MRI of the 5th finger)	
**20/06/2012**	MRI interpretation	tenosynovitis	
**18/07/2012**	Visit to paediatric orthopaedic surgeon	Follow-up, DD: dactylitis	Ibuprofen 200 mg 3x1 for 2 weeks
**01/08/2012**	Visit to paediatric orthopaedic surgeon	Follow-up	Ibuprofen 200 mg 2x1
**19/09/2012**	Visit to paediatric orthopaedic surgeon	Follow-up	Ibuprofen 200 mg 3x1, Controloc 20 mg, observation
**29/10/2012**	Visit to allergy and immunology department.<br>Visit to dermatology	Initial visit, Diagnosis: tenosynovitis	Serology testing for Borrelia burgdorferi, consultation of paediatrics orthopaedic surgeon
**08/11/2012**	Visit to plastic, reconstructive and aesthetic surgeon	Initial visit	MRI follow-u suggested
**21/11/2012**	B. burgdorferi IgM and IgG testing	Negative results (CLIA method)	
**13/05/2013**	Visit to paediatrics endocrinology and diabetes specialist	Initial visit, Differential diagnosis: Proteus syndrome	consultation of paediatrics orthopaedic surgeon
**24/05/2013**	Visit to paediatric haematology and oncology specialist	Initial visit	Skeletal scintigraphy suggested, consultation of paediatrics immunorheumatologist
**06/07/2013**	Visit to paediatrics immunorheumatologist	Initial visit, musculoskeletal ultrasound examination Differential diagnosis: Tenosynovitis or spondyloarthropathy	Indomethacin 25 mg 2x1 per os., Intra-articular triamcinolone hexacetonid injections
**20/08/2013**	Visit to paediatric immunorheumatologist	Follow-up visit; musculoskeletal ultrasound	
**06/12/2013**	Radiology department	Hand radiograms, Differential diagnosis: benign neoplasm of bone and articular cartilage	Consultation of paediatrics orthopaedic surgeon

**14/01/2014**	Visit to paediatric orthopaedic surgeon	Initial visit, Blood work (SR, CBC, CRP), MRI, Skeletal scintigraphy	
**22/01/2014**	Oncology and nuclear medicine department	Skeletal scintigraphy, Differential diagnosis: spondyloarthropathy, RA	
**15/04/2014**	MRI and interpretation	Differential diagnosis: Osteomyelitis, enchondroma, osteoid osteoma	
**13/05/2014**	Visit to paediatric orthopaedic surgeon	Follow-up	
**18/06/2014**	Visit to paediatric orthopaedic surgeon	Follow-up	
**01/07/2014**	Visit to paediatric orthopaedic surgeon	Follow-up	
**10/07/2014**	Visit to anaesthesiology reanimatology and intensive care specialist	Preoperative evaluation (CBC, SR, APTV, PV, TV, fibrinogen, urine)	Consultation of paediatric cardiologist
**10/07/2014**	Visit to paediatric orthopaedic surgeon	Follow-up<br>Hand radiograms	
**11/07/2014**	Visit of paediatric cardiologist	ECG, echocardiography	
**14/07/2014**	Admission to Orthopaedic Division		
**15/07/2014**	Surgery (complete excisional biopsy)		Pathology analysis, clindamycin 3x500 mg i.v. then continuation with 4x300 mg per os
**17/7/2014**	Discharge from Hospital		Clindamycin 4x300 mg per os
**20/07/2014**	Visit to paediatric orthopaedic surgeon	Follow-up;<br>Wound dressing	Clindamycin 4x300 mg per os
**25/07/2014**	Pathology department	Pathology report: osteoid osteoma	
**26/07/2014**	Visit to paediatric orthopaedic surgeon	Follow-up;<br>Wound dressing	Clindamycin 4x300 mg per os excluded
**26/08/2014**	Visit to paediatric orthopaedic surgeon	Follow-up	
**26/04/2015**	Visit to paediatric orthopaedic surgeon	Follow-up	
**26/06/2018**	Visit to paediatric orthopaedic surgeon	Follow-up	
